# Advancing the global physical activity agenda: recommendations for future research by the 2020 WHO physical activity and sedentary behavior guidelines development group

**DOI:** 10.1186/s12966-020-01042-2

**Published:** 2020-11-26

**Authors:** Loretta DiPietro, Salih Saad Al-Ansari, Stuart J. H. Biddle, Katja Borodulin, Fiona C. Bull, Matthew P. Buman, Greet Cardon, Catherine Carty, Jean-Philippe Chaput, Sebastien Chastin, Roger Chou, Paddy C. Dempsey, Ulf Ekelund, Joseph Firth, Christine M. Friedenreich, Leandro Garcia, Muthoni Gichu, Russell Jago, Peter T. Katzmarzyk, Estelle Lambert, Michael Leitzmann, Karen Milton, Francisco B. Ortega, Chathuranga Ranasinghe, Emmanuel Stamatakis, Anne Tiedemann, Richard P. Troiano, Hidde P. van der Ploeg, Juana F. Willumsen

**Affiliations:** 1 Department of Exercise and Nutrition Sciences, Milken Institute School of Public Health, The George Washington University, Washington, DC, USA; 2 Health Promotion Center, Riyadh, Kingdom of Saudi Arabia; 3 Centre for Health Research, University of Southern Queensland, Springfield, Australia; 4 Age Institute, Helsinki, Finland; 5 Public Health Evaluation and Projection Unit, Finnish Institute for Health and Welfare, Helsinki, Finland; 6 Physical Activity Unit, Department of Health Promotion, World Health Organization, Geneva, Switzerland; 7 School of Human Sciences, The University of Western Australia, Perth, Australia; 8 College of Health Solutions, Arizona State University, Phoenix, USA; 9 Department of Movement and Sports Sciences, University of Ghent, Ghent, Belgium; 10 UNESCO Chair, Institute of Technology Tralee, Tralee, Ireland; 11 Department of Pediatrics, University of Ottawa, Healthy Active Living and Obesity Research Group, Children’s Hospital of Eastern Ontario Research Group, Ontario, Canada; 12 School of Health and Life Sciences, Institute for Applied Health Research, Glasgow Caledonian University, Glasgow, UK; 13 Departments of Medicine, and Medical Informatics & Clinical Epidemiology, Oregon Health & Science University, Portland, Oregon USA; 14 MRC Epidemiology Unit, School of Clinical Medicine, University of Cambridge, Cambridge, UK; 15 Baker Heart and Diabetes Institute, Melbourne, VIC 3004 Australia; 16 Diabetes Research Centre, University of Leicester, Leicester General Hospital, Leicester, UK; 17 Department of Chronic Disease and Ageing, Norwegian Institute of Public Health, Department of Sport Medicine, Norwegian School of Sport Science, Oslo, Norway; 18 Division of Psychology and Mental Health, University of Manchester, Manchester, UK; 19 NICM Health Research Institute, Western Sydney University, Sydney, Australia; 20 Department of Cancer Epidemiology and Prevention Research, Cancer Control Alberta, Alberta Health Services, Calgary, Canada; 21 Centre for Public Health, Queen’s University Belfast, Belfast, UK; 22 Department of Non-Communicable Diseases, Ministry of Health, Nairobi, Kenya; 23 Centre for Exercise Nutrition and Health Sciences, School for Policy Studies, University of Bristol, Bristol, UK; 24 Population and Public Health Sciences, Pennington Biomedical Research Center, Baton Rouge, LA USA; 25 Research Centre for Health through Physical Acivity, Lifestyle and Sport, Division of Exercise Science and Sports Medicine, Faculty of Health Sciences, University of Cape Town, Cape Town, South Africa; 26 Department of Epidemiology and Preventive Medicine, University of Regensburg, Regensburg, Germany; 27 Norwich Medical School, Faculty of Medicine and Health Sciences, University of East Anglia, Norwich, UK; 28 PROFITH (PROmoting FITness and Health through physical activity) research group, Department of Physical Education and Sports, Faculty of Sport Sciences, Research Institute of Sport and Health, University of Granada, Spain 26. Sports and Exercise Medicine Unit, Faculty of Medicine, University of Colombo, Colombo, Sri Lanka; 29 Charles Perkins Centre, University of Sydney, School of Health Sciences, Faculty of Medicine and Health, University of Sydney, Sydney, Australia; 30 Institute for Musculoskeletal Health, The University of Sydney, and Sydney Local Health District, Sydney, Australia; 31 Epidemiology and Genomics Research Program, National Cancer Institute, Bethesda, USA; 32 Department of Public and Occupational Health, Amsterdam Public Health Research Institute, Amsterdam UMC, Vrije Universiteit Amsterdam, Amsterdam, The Netherlands; 33 Sydney School of Public Health, The University of Sydney, Sydney, Australia

**Keywords:** Physical activity, Sedentary behavior, Research, Recommendations

## Abstract

**Background:**

In July, 2019, the World Health Organization (WHO) commenced work to update the 2010 Global Recommendations on Physical Activity for Health and established a Guideline Development Group (GDG) comprising expert public health scientists and practitioners to inform the drafting of the 2020 Guidelines on Physical Activity and Sedentary Behavior. The overall task of the GDG was to review the scientific evidence and provide expert advice to the WHO on the amount of physical activity and sedentary behavior associated with optimal health in children and adolescents, adults, older adults (> 64 years), and also specifically in pregnant and postpartum women and people living with chronic conditions or disabilities.

**Methods:**

The GDG reviewed the available evidence specific to each sub-population using systematic protocols and in doing so, identified a number of gaps in the existing literature. These proposed research gaps were discussed and verified by expert consensus among the entire GDG.

**Results:**

Evidence gaps across population sub-groups included a lack of information on: 1) the precise shape of the dose-response curve between physical activity and/or sedentary behavior and several of the health outcomes studied; 2) the health benefits of light-intensity physical activity and of breaking up sedentary time with light-intensity activity; 3) differences in the health effects of different types and domains of physical activity (leisure-time; occupational; transportation; household; education) and of sedentary behavior (occupational; screen time; television viewing); and 4) the joint association between physical activity and sedentary time with health outcomes across the life course. In addition, we acknowledge the need to conduct more population-based studies in low- and middle-income countries and in people living with disabilities and/or chronic disease, and to identify how various sociodemographic factors (age, sex, race/ethnicity, socioeconomic status) modify the health effects of physical activity, in order to address global health disparities.

**Conclusions:**

Although the 2020 WHO Guidelines for Physical Activity and Sedentary Behavior were informed by the most up-to-date research on the health effects of physical activity and sedentary time, there is still substantial work to be done in advancing the global physical activity agenda.

In July, 2019, the World Health Organization (WHO) convened a group of public health scientists and practitioners to serve on the Guidelines Development Group (GDG) for the 2020 Guidelines on Physical Activity and Sedentary Behavior [1]. The GDG comprised 27 physical activity experts from selected disciplines (epidemiology, physiology, health behavior, etc.), as well as policy makers and end-users of the recommendations and was balanced by global region and by gender. The overall task of the GDG was to review the scientific evidence and provide expert advice to the WHO on the amount of physical activity and sedentary behavior associated with optimal health in children and adolescents, adults, older adults (> 64 years), and also, specifically in pregnant and postpartum women and people living with chronic conditions or disabilities. These 2020 Guidelines (as well as those for children ages 0–5 years) [2] replace the recommendations released by the WHO in 2010 [3], as they include the most current evidence available across a broader life course.

For children and adolescents (aged 5–17 years) the current reviews updated the evidence syntheses conducted for the 2016 Canadian 24-Hour Movement Guidelines for Children and Youth [4–6], the 2019 Australian 24-Hour Movement Guidelines for Children and Young People (5–17 years) [7] and the Physical Activity Guidelines for Americans, 2nd edition that were released in 2018 [8]. For adults, older adults and sub-populations, the reviews updated evidence syntheses conducted for the Physical Activity Guidelines for Americans, 2nd edition [8] and the 2019 Canadian Guidelines for Physical Activity Throughout Pregnancy [9]. Systematic reviews published from 2017 to July 2019 were identified that addressed the key questions, and the Grading of Recommendations Assessment, Development and Evaluation (GRADE) framework was used to rate the certainty of the evidence.

Full details of the methods for identifying the most current evidence to inform these 2020 Guidelines are described in detail elsewhere [10]. While reviewing the available evidence to inform the 2020 Guidelines, the GDG identified a number of gaps in the existing literature, many of which were carried forward from our earlier work on the Australian [7], Canadian [4–6, 9], and United States [8] Guidelines. These proposed research gaps were discussed and verified by expert consensus among the entire GDG. Below, we describe the research gaps according to the different population sub-groups investigated, along with research recommendations that could advance the global physical activity agenda and future public health practice. These research recommendations are listed in Tables 1 and 2**.**

**Table 1:** Research recommendations for children & adolescents, adults, older adults, people with disabilities, and pregnant women

**General Research Recommendations** **(all age groups and sub-populations)**	Conduct RCTs, Mendelian randomization studies, and prospective cohort studies that use device-based measures to address a range of physical activity exposures (volume and/or intensity) and sedentary time, in order to determine the dose-response relationship between these behaviors and a broad range of health outcomes in this age group.
	Conduct adequately-powered experimental studies to examine the health benefits of light-intensity physical activity and of breaking up sedentary time with light-intensity activity.
	Conduct adequately-powered prospective observational studies using device-based measures and self-report to examine differences in the health effects of various types and domains of physical activity (leisure-time; occupational; transportation; household; education) and of sedentary behavior (occupational; class or study time; screen time; television viewing).
	Conduct adequately-powered observational studies to examine the joint association between physical activity and sedentary time with health outcomes across the life course.
**Specific Research Recommendations for Children and Adolescents (5–17 years)**	
	Conduct longitudinal studies of various domains of sedentary behavior (e.g., total sitting time, TV time, video-games, computer/phone screen time) and health, using both self-report and device-based measures that can distinguish between different postures (reclining, sitting, standing, and moving).
	Conduct adequately-powered experimental studies on the effects of interruptions or breaks in sedentary behavior with physical activity of various intensities and durations on health biomarkers, such as blood pressure or blood concentrations of glucose, insulin and lipids.
	Conduct adequately-powered observational studies to examine the independent and joint effects of physical activity and sedentary behavior on health outcomes in children and adolescents.
	Develop standardized (harmonized) methods of measuring and processing device-based estimates of physical activity in this age group.
**Specific Research Recommendations for Adults (≥18 years)**	Conduct adequately-powered population-based studies of adults that include both self-report and device-based measures of physical activity to improve the quantification of domain-specific and type-specific physical activity and to examine their dose-response relationships with various health and disease outcomes.
	Conduct high quality studies examining specific characteristics of occupational physical activity and their effects on worker health.
	Conduct experimental studies to determine if the benefits of physical activity for health differ with regard to muscular strength training vs. aerobic exercise training.
	Conduct adequately-powered population-based studies using pooled analyses, as well as prospectively- or retrospectively-harmonized meta-analyses to examine the role of physical activity in health and function.
	Conduct adequately-powered population-based studies in high-, middle- and low-income countries to compare and contrast the relationships among different types and domains of physical activity and health outcomes.
	Conduct adequately-powered population-based studies to examine the role of physical activity and sport in increasing community cohesion and social capital.
**Specific Research Recommendations for Older Adults (> 64 years)**	Conduct adequately-powered observational and experimental studies to investigate further the dose-response relationships between different intensities, volumes, and types of physical activity (aerobic, muscle strengthening, balance, and multicomponent) and multiple health outcomes. The reporting of adverse events in these studies is especially important for establishing safety thresholds.
	Conduct adequately-powered RCTs of older adults at high risk of falls designed with fall-related injuries and bone fractures as the primary outcomes of interest.
	Conduct adequately-powered RCTs to determine the effects of specific alternative or complementary forms of exercise on the reduced risk of falls and on physical function in healthy older adults, as well as those with different chronic conditions.
	Conduct more experimental research on dual-task training that clearly describe the dual-task training procedures and the parameters of the outcome task. In addition, these studies should provide evidence of whether dual-task benefits were increased by training and whether dual-task training transfers to untrained tasks.
	Conduct adequately-powered RCTs with 6- and 12-month post-intervention follow-up assessments to determine the effects of physical activity on activities of daily living (ADL) mobility, instrumental ADLs, free-living physical/ ambulatory activity and social participation for older individuals with existing chronic disease, who may be at accelerated risk of physical and cognitive decline, disability, and social isolation.
	Conduct adequately-poweredcohort or experimental studies on the effects of specific types of physical activity on perceived social isolation and loneliness.
	Conduct adequately-poweredcohort and experimental studies to determine the dose-intensity and timing of physical activity necessary to prevent functional decline or to improve physical function across the spectrum of cognitive dysfunction and dementia.
**Specific Research Recommendations for People with Disabilities**	Conduct adequately-powered observational and experimental studies that examine the relationship between physical activity, sedentary behavior, and health and wellbeing in people living with intellectual, mental, physical, and/or sensory impairments.
	Conduct adequately-powered RCTs that are targeted toward different types of impairment (e.g. physical, sensory, or cognitive) and different degrees of impairments (from mild to complete), rather than only on specific health conditions such as multiple sclerosis, spinal cord injury, intellectual disability, Parkinson’s disease, or stroke. Include people with disabilities into large “mainstream” studies (from which they are typically excluded) in order to increase the generalizability of findings.
	Conduct mixed-methods studies to examine the physical, social and attitudinal barriers and facilitators to physical activity for people living with disabilities, as well as appropriate policies and strategies to encourage and support participation.
**Specific Research Recommendations during Pregnancy**	Conduct adequately-powered RCTs on the health benefits of breaking up sedentary time with bouts of light-intensity activity.
	Conduct adequately-powered observational research on the joint association of physical activity and sedentary time with maternal health and fetal outcomes.
	Conduct observational and experimental studies of the effects of vigorous-intensity physical activity before and during pregnancy on maternal and fetal outcomes.
	Conduct experimental and observational studies to investigate the effects of various types, intensities, and volumes of regular physical activity on quality of life, sleep, and symptoms of anxiety and depression during pregnancy and the postpartum period.
	Conduct adequately-powered observational studies to determine whether the timing (before, during, or following pregnancy) or specific domains/settings of physical activity affect maternal and fetal outcomes, such as preterm birth, low birth weight, and preeclampsia differentially. These studies should have ample statistical power within the different domains to be able to adjust for the influence of several confounding variables.
	Conduct observational and/or experimental research that has adequate statistical power to determine whether the associations between physical activity and maternal or fetal outcomes vary by age, race/ethnicity, socioeconomic status, or by weight status.

**Table 2:** Research recommendations for people living with cancer, type 2 diabetes, hypertension, and HIV

**Cancer**	Conduct prospective cohort studies of cancer survivors to include cancer sites for which there is limited or no evidence of an association between physical activity and all cancer outcomes (i.e. cancer recurrence, new primary cancers, cancer-specific mortality and all-cause mortality).
	Conduct prospective cohort studies of cancer survivors that include repeated self-report and device-based measures of physical activity to determine the long-term effects of physical activity on cancer outcomes.
	Conduct prospective cohort studies of cancer survivors within understudied populations as defined by race, ethnicity, socioeconomic status, cancer stage (i.e. advanced or metastatic cancers), or cancer treatment (e.g. cardiotoxic drugs, radiotherapy, hormone treatments)
	Conduct prospective cohort studies in cancer survivors that include objective measures of health-related fitness and follow-up for cancer outcomes.
	Conduct randomized controlled intervention trials in cancer survivors to assess the impact of physical activity on cancer outcomes. Trials should include assessments of different domains, types, and doses of physical activity and their impact on specific cancer types.
**Type 2 Diabetes**	Conduct studies that include both self-report and importantly, device-based measures of physical activity and sedentary time to determine whether measurement modality influences associations with health outcomes, particularly co-morbid conditions, disease progression indicators, physical function, and health related quality of life.
	Conduct RCTs comparing the effects of shifting time from specific forms of sedentary behavior to low-intensity aerobic activity, moderate-intensity aerobic activity, low-intensity muscle-strengthening activity, and moderate-intensity muscle-strengthening activity on indicators of risk of progression of type 2 diabetes.
	Conduct further systematic and coordinated RCTs on the health effects of tai chi, qigong, and yoga in people with type 2 diabetes to improve this emerging evidence base.
	Conduct research on whether or not individual characteristics (e.g. sex, disease progression) influence the effects of physical activity interventions on health outcomes in people with type 2 diabetes.
**Hypertension**	Conduct studies with greater homogeneity in population characteristics across the studies included in systematic reviews (i.e., exclusively adults with hypertension) to strengthen the evidence on the association between physical activity and comorbid conditions, physical function, health-related quality of life, and disease progression.
	Conduct prospective cohort studies of adults with hypertension using device-based measures of physical activity to determine the dose-response relationship between physical activity and disease progression outcomes.
	Conduct prospective cohort studies of adults with hypertension within understudied populations as defined by race, ethnicity, socioeconomic status, and disease progression.
	Conduct prospective cohort studies in adults with hypertension that include objective measures of physical function and ratings of health-related quality of life.
	Conduct RCTs in adults with hypertension to assess the impact of physical activity on disease progression outcomes. Trials should include assessments of different domains, types, and doses of physical activity and their impact on disease progression.
**People Living with HIV**	Conduct studies on the association between physical activity and health outcomes in people living with HIV living in low- to moderate-income countries.
	Conduct RCTs with intention-to-treat analyses to address high attrition and reduce heterogeneity between studies.
	Conduct RCTs testing different types and doses of exercise on health outcomes in people living with HIV.
	Conduct studies using both self-report and device-based measures of physical activity and sedentary behaviors to improve the quantification of these behaviors.
	Conduct studies that test directly the potential interactions between physical activity and the highly active anti-retroviral therapy on health outcomes such as body composition, cardiometabolic risk and disease progression.

## Children and adolescents (5–17 years)

Although there is evidence of a dose-response relationship between physical activity and health outcomes during childhood and adolescence, evidence is currently insufficient to determine an optimal physical activity dose. Many of the health benefits are observed with an average of 60 min/day of moderate-to-vigorous physical activity (MVPA); however, MVPA beyond 60 min/day appears to be even better for specific health outcomes, such as reduced adiposity. Few studies have been designed specifically to examine (in a prospective manner) the dose-response relationship between physical activity and specific health outcomes in children and adolescents, and the shape of the dose-response curve between sedentary behavior and health outcomes has not been examined at all in this age group. Such studies will help provide further details on the optimal volume, duration, frequency, and intensity of physical activity and the sedentary time thresholds necessary for maximal health benefits at different stages of childhood and adolescence. Similarly, there is a lack of robust scientific evidence in children on the potential health benefits of breaking up prolonged periods of sitting on various health biomarkers, such as blood pressure or blood concentrations of glucose, insulin and lipids. Again, this information is key for quantifying the optimal combination of frequency, intensity and duration of such interruptions to inform future public health guidelines. The new 2020 WHO Guidelines [1, 10] also do not specifically mention light-intensity physical activity for children and adolescents. From a public health perspective, light-intensity activity is important because more people would be likely to do this type of activity for longer periods of time, compared with moderate- or vigorous-intensity activity. Thus, increasing knowledge about the benefits of light-intensity activity in children is key to understanding the extent to which substituting sedentary behaviors with light-intensity physical activities impacts health. Device-based measurements of physical activity and sedentary time are ideal, as they now can measure accurately a range of activity intensities that was not possible when relying on self-report alone [11]. Finally, whereas most studies have examined physical health outcomes such as adiposity, physical fitness and cardiometabolic health biomarkers [4–8], it is also important to strengthen the knowledge across a broader range of health and functional outcomes, such as musculoskeletal health, mental health, cognition, immunity, academic achievement, quality of life, optimal physical and emotional growth, and motor skill development. Therefore, there is a need for randomized controlled trials (RCTs) and prospective cohort studies that use device-based measures to address a range of physical activity exposures (volume and/or intensity) and sedentary time, in order to determine the dose-response relationship between these behaviors and a broad range of health outcomes in children and adolescents.

Evidence is also lacking to determine if the association between physical activity and health outcomes in children and adolescents varies by type (e.g., aerobic vs. muscle-strengthening), domain (e.g., leisure-time vs. physical education vs. transportation), or location (outdoor vs. indoor) of physical activity. The GDG identified emerging evidence suggesting that physical activity of any type is more beneficial when performed outdoors “in nature” than when the same activity is performed indoors [12]. In addition, the health benefits of play (i.e., unstructured physical activity) are being recognized for people of all ages [13]. However, there was insufficient evidence on aspects of this domain of activity in youth to provide any detailed specification in the new 2020 guidelines. A better understanding of the potentially distinct and beneficial effects of physical activity of different types, domains, and locations may be important for more targeted and flexible intervention strategies and to inform future guidelines.

Very few studies that examined the association between sedentary behavior and health outcomes in children have relied on device-based measures [4–8]. Furthermore, many studies have focused only on self-report of television viewing/screen time as the behavioral marker of sedentary time [4–8]. New technology (smart phones and tablets, live-streaming) is changing both the duration and nature (binge-watching while reclining in bed) of sedentary behaviors. Newer device-based measures [14] can distinguish between different postures (reclining, sitting, standing, and moving) and such research could allow us to identify a spectrum of different sedentary behaviors in children and adolescents that negatively impact health.

With regard to device-based measures of physical activity, it is important to note the large degree of variability in these methods (e.g., type and model of device, placement on the body, cut-points used to define different intensities, analytical approaches, etc.). Standardized (i.e., harmonized) methods are needed to provide more reliable estimates of physical activity and sedentary behavior that are comparable across different pediatric study populations.

Finally, there is evidence in adults [15] and older adults [16] that the health impact of sedentary behavior (particularly television viewing) becomes especially detrimental when combined with low levels of physical activity. Thus, the association between physical activity and specific health outcomes in children and adolescents may also be modified by the amount of sedentary behavior they engage in. This effect modification by sedentary behavior suggests that physical activity levels greater than the recommended threshold of ≥60 min/day of MVPA may be needed to lower the risk of weight gain or cardiometabolic impairments among youth who spend large amounts of time sitting or reclining. On the other hand, adolescents who engage in vigorous sport activity may need sedentary time for rest and recovery. Currently, there is no prospective information on the joint effects of physical activity and sedentary behavior on health outcomes in children and adolescents. This information is key for future guidelines on the optimal combination of these behaviors for health and function throughout childhood and adolescence.

### Adults

Several research gaps identified for children and adolescents are also relevant for adults (e.g., the shape of the dose-response curve, the combined associations of physical activity and sedentary behavior with health outcomes, and the benefits of breaking up sedentary time with light-intensity activity). Adults, however, engage in physical activity of various intensities across multiple domains (e.g., transportation, occupational, household, and leisure-time). Self-report methods alone cannot capture the many health-related aspects of physical activity, and this lack of complete evidence is particularly true for short bouts of lower-intensity activity that may be highly-variable, but contribute markedly to the total volume of daily energy expenditure [17]. Conversely, device-based measures do not capture the specific type or domain of physical activity in which measured step- or activity-counts are accumulated. Few studies currently employ both methods of assessment (i.e., self-report and device-based), which is important for establishing the dose-response relationship between different domains of physical activity and health outcomes more accurately and comprehensively. More use of combined methods therefore is warranted.

While there has been considerable progress since 2010, much of the evidence on the beneficial effects of physical activity still comes from high-income countries and so more research is needed from low- and middle-income countries (LMIC). In particular the GDG identified the importance of more research on the different physical activity patterns, which for large sectors of the populations in LMIC comprise low- and moderate-intensity occupational physical activity performed over the majority of the day [18]. Knowledge of the relationship between this pattern of labor-related activity and well-being could help to address global health disparities – and this is particularly relevant for women of reproductive age, who often carry a dual burden of outside labor and family care activities [18]. In high-income countries, the health effects of occupational physical activity are equivocal and the data may be confounded by socioeconomic status, discrimination, exposure to hazardous work conditions, or (as is the case in the United States) limited access to health care. Moreover, the optimal balance between occupational activity and sedentary behavior over the course of the workday has not been established. Such information could inform work-site policies to improve occupational health among all workers.

Currently, few observational studies use pooled analyses or prospectively- or retrospectively-harmonized meta-analyses to address complex research questions. Meta-analyses using harmonized data are more economical and provide superior statistical power to that of an individual cohort study. Pooled analyses and harmonized meta-analyses can examine detailed research questions with higher precision, and likely, with broader internal and external validity.

Finally, promoting a more physically active society benefits our collective physical and mental well-being; however, the influence of physical activity and sport on building social capital among different communities has not been studied in a rigorous manner. Indeed, sport-for-development and peace (SDP) efforts, which use physical activity, sport, and game-based programming to address specific development and peace initiatives to empower individuals and communities [19], are relatively new to public health. Enhanced social capital can, in turn, influence the policies necessary for making the environmental changes for increasing population-levels of physical activity [20]. Further evaluation of SDP initiatives is needed to guide the best use of limited resources and maximize program effectiveness.

### Older adults (> 64 years)

The lack of knowledge about dose-response, the combined association of physical activity and sedentary behavior with health, and the benefits of breaking-up sedentary time with light-intensity physical activity that were identified as research gaps for children, adolescents, and adults remain especially relevant for those over age 64 years; however, a number of research needs that are specific to this age group were identified. For example, although the effects of the *minimal effective dose* of physical activity (150 min/week of moderate-intensity activity) on health and function have already been described for older people [21], we have yet to examine greater volumes of activity in order to establish *maximal safety thresholds.* This issue is particularly relevant for older people having pre-existing chronic conditions or limitations [22]. Because physiologic reserve declines with advancing age, studies need to examine several relative and absolute levels of physical activity intensity, and the monitoring and reporting of adverse events remains important for establishing safety thresholds.

The age-standardized incidence of falls world-wide was 2238/100,000 in 2017 [23]. These falls are the leading cause of fatal injury and the most common cause of non-fatal trauma-related hospital admissions in this age group [23]. Unfortunately, many studies are designed to examine the etiologic relation between physical activity and the risk or rate of falling, with fall-related injuries or bone fractures being a secondary outcome. This is a problem resulting in insufficient sample sizes, which often preclude our ability to determine the risk of specific types of fall-related injuries, as well as the behavioral, sociodemographic, and environmental determinants of those injuries. There is thus a need to conduct adequately-powered epidemiologic studies or RCTs specifically to examine fall-related injuries among older adults most susceptible to falling (e.g., those with balance or mobility impairments and those with frailty).

Tai chi, Qigong, dance, active video gaming, and yoga are alternative and complementary forms of exercise that are now recognized as effective approaches for maintaining and improving overall physical function, as well as balance and gait in older people [21, 22]. Furthermore, the benefits of rigorous multicomponent activities (i.e., those that combine aerobic, strength, and balance training), as well as novel interventions that integrate “functional exercises” into everyday tasks, need to be examined further. For older people with prevailing chronic disease and/or mobility limitations, these sorts of activities may be especially beneficial. Thus, research on such physical activities should consider not only the minimal effective doses for improving physical function, but also the types or modes of such activity that are most effective for specific chronic conditions.

A relatively new area of aging research concerns that of dual-task training (i.e., the combination of a physical task with a cognitive task). To date, however, the methodologic quality of existing studies ranges from poor to moderate [21]. The evidence generally provided inadequate descriptions of the dual-task training protocol itself and only considered one outcome task (such as balance or a cognitive task) that may also have been part of the dual-task training. Future experimental studies should include multiple outcome tasks -- some that are part of the training protocol, as well as some novel (untrained) outcome tasks -- to examine whether such training enhances dual-task benefits and whether untrained tasks can also benefit from such training.

It is not entirely clear how physical activity interventions designed to improve aspects of physical function (strength, balance, flexibility, and endurance) translate into general improvements in activities of daily living (ADLs), not to mention advanced- and instrumental-ADLs that influence social participation -- and this is especially so after the formal intervention period ends. This knowledge is key to understanding how enhanced physiologic function can influence certain behavioral aspects of healthy aging (self-care, independence, social engagement), as well as quality of life for older people. Older adults with existing chronic disease may be at accelerated risk of physical and cognitive decline, thus leading to disability, and social isolation. Thus, adequately-powered RCTs with follow-up assessments of at least 6- to 12-months are recommended to determine the effects of improvements in physical function on changes in the spectrum of ADL function and social participation. Importantly, social isolation and loneliness are now regarded as one of the major risks to health and quality of life in older age, even among those who are fully functional [24]. Yet, there is very little evidence of the effects of physical activity on this important psychosocial factor.

Finally, physical function limitations often exist simultaneously with cognitive dysfunction and dementia; yet, there is limited evidence about the impact of physical activity training on concurrent improvements in physical and cognitive function [21]. Since mobility and cognition are closely linked, our ability to improve physical function in a cognitively impaired population (using dual-task training, for example) could have broad implications for independence and quality of life in older people. Thus, there is a need for adequately-powered cohort and experimental studies to determine the dose, type, and timing of physical activity necessary to maintain or to improve physical function across the spectrum of cognitive dysfunction and dementia.

### Pregnant and postpartum women

The period of gestation is an important time to engage in health behaviors that can have both transient and long-lasting benefits for the mother and baby. Some of the research gaps identified for other populations remain especially relevant for pregnant women (e.g., the health benefits of breaking up sedentary time with bouts of light-intensity activity, or the joint association of physical activity and sedentary time with maternal and fetal health). On the other hand, although the myriad benefits of moderate-intensity physical activity during pregnancy and the postpartum period have been established [9, 21, 25], the safety and benefits of vigorous-intensity physical activity have been examined only recently [26]. Women who participate in vigorous physical activity on a regular basis before pregnancy may want to continue such activity for as long as possible throughout pregnancy and the post-partum period. Therefore, studies that examine the effects of vigorous-intensity (performed before, during, and post-pregnancy) would provide important information on minimal effective levels of vigorous activity, as well as maximal threshold levels for safety issues such as hyperthermia, low gestational weight gain, musculoskeletal injury, or low birth weight [25].

While there is evidence that regular, moderate-intensity physical activity reduces symptoms of post-partum depression, [9, 21, 25] we know little about the relationship between physical activity and depression, anxiety, quality of life, or sleep quality during the gestational period itself. There is some evidence that the health of the developing fetus is intrinsically linked to maternal mental health [27]. Thus, knowledge about the benefits of various doses and modes of physical activity to maternal mental health can serve to promote a healthy pregnancy for both mother and offspring.

Most of the experimental research on physical activity and health among pregnant women relies on a standard dose of 150 min/week of moderate-intensity aerobic activity [21]. Therefore, information is limited about the health effects of other types (e.g. muscle strengthening activity) or doses of physical activity that are performed before, during, or following the gestational period. Moreover, there is some evidence that activities performed in the occupational setting (such as prolonged standing or lifting heavy loads) have health consequences for pregnant women that are different than when these same activities are performed during leisure time [28]. Presumably, these differences in health effects are related to the inability to take breaks from strenuous activity at the work site, as well as to the confounding influences of socioeconomic status, education level, and age. Thus, knowledge about the impact of varying types and doses of physical activity that are performed within different domains (leisure-time, occupational, household, transportation) enhances our ability to inform both clinical and public health practice.

Finally, most studies examining the impact of physical activity on health during pregnancy lack the statistical power to test whether the results vary by sociodemographic characteristics (e.g., age, race/ethnicity, educational attainment) or by body weight. Moreover, most of the evidence comes from high-income countries and therefore, little is known about how the dual burden of occupational labor and childcare affects maternal health or how cultural factors may modify this association. The reduction of health disparities among pregnant women is an important global public health consideration. Therefore, studies that are adequately-powered to test for effect modification by sociodemographic or other sociocultural factors can help to inform more specific physical activity guidelines for different population sub-groups of women.

### People with disabilities

The GDG concluded that more research was needed among people living with disability across all ages –especially with regard to existing comorbid conditions and the risk of the major non communicable diseases. This research is particularly important for those with certain intellectual, mental, mobility, or sensory disabilities/impairments who may spend more than the average amount of time sitting or reclining and therefore, may benefit the most from physical activity interventions for better health, functioning and participation. As there is much variation in disability levels among people with the same health condition, it is important to match specific types of physical activity to the specific needs, abilities, and interests of individuals in order for them to get the maximal enjoyment and health benefits. Thus, RCTs are needed that are targeted toward different types of impairment (e.g. physical, sensory, or cognitive) and different degrees of impairments (from mild to complete), rather than only on specific health conditions such as multiple sclerosis, spinal cord injury, intellectual disability, Parkinson’s disease, or stroke. Another approach is to recruit people with disabilities into large “mainstream” studies (from which they are typically excluded) in order to increase the generalizability of findings.

Finally, an active lifestyle is a basic human right for all of society. People with disabilities, however, may experience difficulties engaging in regular physical activity due to a number of environmental and social barriers. Public policy for promoting physical activity is lagging behind other health promotion policies, and this gap is particularly large for people with disabilities. Mixed method studies could provide the knowledge and understanding of the barriers and facilitators of physical activity for people with disabilities to inform local, regional, national, and global policies that call for inclusivity and that support participation in physical activity and sport for all.

### People living with chronic conditions

Approximately 33% of adults globally suffer from two or more chronic diseases, and this burden is greater in low- compared with high-income countries [29]. Again, many of the research gaps identified for adults and older adults are also pertinent for people who are living with chronic conditions. The issue of ensuring adequate sample sizes in order to study effect modification of the relationship between physical activity or sedentary behavior and chronic disease progression by age, race, sex, and income remains important for reducing health disparities. The use of both device-based and self-reported measures of physical activity and sedentary behavior to establish dose-response curves for these behaviors occurring across different domains of activity are key for establishing minimal effective dose and maximal safety thresholds to support health and function in those living with one or more chronic disease(s). People with chronic disease are more likely to be sedentary. As was the case for children, adults, and older adults, studies that provide evidence about the effects of shifting time from sitting to light- or moderate-intensity activity are key to understanding how even short bouts of various levels of physical activity can influence disease progression and other health outcomes, such as physical and cognitive function and quality of life. Finally, the need to conduct pooled analyses, as well as prospectively- or retrospectively-harmonized meta-analyses of epidemiologic studies in adults and children with chronic conditions remains important to examine detailed etiologic relationships with greater internal and external validity.

The GDG also identified several research gaps that are specific to those with chronic conditions – with special relevance for those who are cancer survivors and for people living with HIV. To date, very few well-designed epidemiologic studies or RCTs have been conducted in these two groups. For example, prospective cohort studies are needed among cancer survivors for cancer sites other than breast and colon cancer that have not been sufficiently investigated to date, as well as studies in advanced or metastatic cancers. Studies also need to examine more fully the influence of cancer treatment (e.g. cardiotoxic drugs, radiotherapy, hormone treatments) to determine whether the role of physical activity and sedentary behavior on disease progression (remission, recurrence, new primary cancers or mortality) varies by these factors. Similarly, research is needed to consider how physical activity interacts with the highly active anti-retroviral therapy to affect body composition, cardiometabolic risk and, importantly, disease progression in people living with HIV. Since more than 37 million people world-wide currently are living with HIV [30], this information has enormous public health significance. Table 2 provides a full list of research recommendations for the chronic conditions of cancer, diabetes, hypertension, and HIV.

In summary, although the *2020 WHO Guidelines for Physical Activity and Sedentary Behavior*^*1*^ were informed by the latest available research on the health effects of physical activity and sedentary behavior, additional work is needed to advance the global physical activity agenda. Evidence of the health benefits of regular physical activity has increased exponentially over the past several decades (with notable gaps in the area of disability); yet globally, about 28% of adults ≥18 years of age and 81% of adolescents aged 11–17 years still do not meet the WHO recommendations for physical activity [31, 32]. Thus, evidence-based research targeted toward individuals needs to be complimented with efforts geared toward raising population levels of physical activity. For example, little is known about the social determinants that drive population physical activity adoption and maintenance within different countries and a better understanding is needed of the social return on investment (i.e., the combined economic, social, and environmental benefits) in population physical activity promotion programs. Promoting population physical activity requires us to “scale-up” effective interventions that have outgrown research dependency and embed them into the “whole of society” (e.g., government, education, health care, transportation, urban design and architecture) so that they can reach all citizens in a given community [33].

Currently, the evidence on the effectiveness of large scale and national initiatives to promote population physical activity is minimal [33], and evaluating their effectiveness at the population level is a challenge. Moreover, the research-to-public health practice time gap can be as long as 17 years [34], and closing this gap traditionally has not been a priority in academic research [35]. The field of implementation science evolved in order to facilitate the “scale-up” and “speed-up” of evidence-based interventions and practices into common practice and policies within communities, with the goal of improving population health [36]. In order to scale-up effective evidence-based physical activity programs, however, we need to identify key stakeholders across multiple levels of society and work through key barriers to implementation in “real world” environments [37]. Indeed, these methods shift our thinking from a reliance on evidence-based practice toward that of practice-based evidence (i.e., what actually works at the population level). As this field continues to grow and becomes a funding priority, new research opportunities will emerge and new investigators will be trained who are better able to translate public health science into public health practice for all, in order to advance our global physical activity agenda.

## Data Availability

The materials and evidence used for the current manuscript are available from the corresponding author on reasonable request.

## Abbreviations

ADL: Activities of Daily Living
GDG: Guidelines Development Group
HIV: Human Immunodeficiency Viruses
MVPA: Moderate-to-Vigorous Intensity Physical Activity
PAGAC: Physical Activity Guidelines Advisory Committee
RCT: Randomized Controlled Trial
SDP: Sport for Development and Peace
WHO: World Health Organization
